# The Interplay Between Emotion Dysregulation and Repetitive Thoughts in Insomnia Disorder: The Impact of Worry, Rumination and REM Sleep Instability

**DOI:** 10.1111/jsr.70267

**Published:** 2025-12-14

**Authors:** Samantha Mombelli, Elisabetta Fasiello, Maria Caterina Di Perri, Francesca Casoni, Marco Zucconi, Luigi Ferini‐Strambi, Andrea Galbiati

**Affiliations:** ^1^ Center for Advanced Research in Sleep Medicine Centre Intégré Universitaire de Santé et de Services Sociaux du Nord de l'Île‐de‐Montréal Montréal Québec Canada; ^2^ Department of Psychiatry and Addictology Université de Montréal Montréal Québec Canada; ^3^ Department of Clinical Neurosciences Neurology‐Sleep Disorders Center, IRCCS San Raffaele Scientific Institute Milan Italy; ^4^ Sleep Medicine Center, Neurophysiopathology and Movement Disorders Unit, Department of Clinical and Experimental Medicine University of Messina Messina Italy; ^5^ Faculty of Psychology Vita‐Salute San Raffaele University Milan Italy

**Keywords:** emotion dysregulation, insomnia, REM sleep, rumination, worry

## Abstract

While previous research has identified emotional dysregulation and repetitive thinking as contributors to insomnia, the interplay between these factors remains unclear. Building upon data previously collected in our laboratory, this exploratory study extends prior findings by examining the mediating role of rumination, worry and rapid eye movement (REM) sleep instability in the relationship between emotional dysregulation and the functional impact of insomnia and depressive symptoms, aiming to generate hypotheses about the psychological and neurophysiological mechanisms linking these constructs. Using the same cohort of 23 patients with insomnia disorder and 23 matched healthy sleepers, participants underwent overnight polysomnography and completed validated questionnaires assessing emotional dysregulation, worry, rumination and the functional impact of insomnia. Novel mediation models were used to examine whether worry, rumination and REM sleep instability mediated the link between emotional dysregulation and daytime consequences of insomnia. Compared to controls, individuals with insomnia showed significantly greater emotional dysregulation, rumination and worry. Mediation analysis indicated that rumination, but not worry, significantly mediated the relationship between emotional dysregulation and daytime consequences of insomnia. Furthermore, higher scores on the dimension ‘difficulties in distracting with emotions’ were associated with increased REM sleep instability, which also mediated the effect of emotional dysregulation on daytime consequences of insomnia. These findings highlight the crucial role of rumination in sustaining the functional impact of insomnia and suggest that interventions targeting repetitive negative thinking and emotional regulation may improve sleep outcomes.

## 1 Introduction

Insomnia disorder is among the most prevalent sleep disorders, affecting a substantial portion of the global population (Wittchen et al. 2011). It is characterised by difficulties initiating or maintaining sleep, early‐morning awakenings or nonrestorative sleep, despite adequate opportunity and circumstances for rest (American Academy of Sleep Medicine 2024). Chronic insomnia is associated with impairments in daytime functioning, including cognitive deficits, emotional distress and an elevated risk of developing mood and anxiety disorders (Hertenstein et al. 2019). While physiological and behavioural factors contribute to the onset and maintenance of insomnia, a growing body of research highlights the critical role of emotional and cognitive processes in perpetuating sleep disturbances (Baglioni et al. 2024; Fernández‐Mendoza et al. 2010). In particular, emotional dysregulation, worry and rumination have been identified as key psychological mechanisms implicated in the chronicity of insomnia disorder (Galbiati et al. 2020; Jansson and Linton 2006; Riemann et al. 2025; Watkins and Roberts 2020). However, the complex interplay among these factors remains insufficiently understood.

Emotional dysregulation refers to difficulties in modulating emotional responses, which may result in heightened emotional reactivity and the use of maladaptive regulation strategies (Gross 2013). Individuals with insomnia frequently report intense negative emotions, such as frustration, anxiety and sadness, in response to sleep difficulties (Olatunji et al. 2023). These heightened emotional responses can, in turn, exacerbate sleep disturbances by increasing physiological arousal and cognitive hyperactivity, both considered core mechanisms underlying insomnia (Kalmbach, Anderson, et al. 2018; Kalmbach, Cuamatzi‐Castelan, et al. 2018; Zhao et al. 2024). Moreover, maladaptive regulation strategies, such as emotional suppression, avoidance or excessive worry, can impair the ability to manage stress and negative affect, further reinforcing sleep problems (Ballot et al. 2021). Importantly, emotional dysregulation has also been linked to disruptions in rapid eye movement (REM) sleep, a critical sleep stage involved in emotional processing and memory consolidation. Increased arousal during REM sleep, often influenced by repetitive negative thought patterns, may contribute to fragmented sleep and difficulty achieving restorative rest (Feige et al. 2023). Worry and rumination are two cognitive processes closely linked to emotional dysregulation and extensively implicated in insomnia. Worry is a repetitive and uncontrollable thought process focused on future threats and uncertainties, often associated with anxiety disorders (Borkovec et al. 1983). In the context of insomnia, individuals frequently experience pre‐sleep worry, anticipating negative consequences of sleep loss and fearing an inability to function the next day (Gerlach et al. 2020). This cognitive activity can contribute to heightened physiological arousal, making it difficult to transition into sleep (Lemyre et al. 2020). Research has shown that excessive worry is associated with increased sleep latency (SL), frequent nocturnal awakenings and overall poorer sleep quality (Galbiati et al. 2018; Lancee et al. 2017). On the other hand, rumination involves repetitive, passive thinking about past events, perceived failures or distressing emotional experiences (Nolen‐Hoeksema 2000; Nolen‐Hoeksema et al. 2008). Commonly observed in individuals with depression, rumination has been associated with sustained negative mood states (Zhou et al. 2020). In insomnia, rumination often manifests as persistent reflections on prior nights of poor sleep, self‐critical thoughts or distress regarding past stressors (Kalmbach, Anderson, et al. 2018; Kalmbach, Cuamatzi‐Castelan, et al. 2018). This negative self‐focus can delay sleep onset and prolong nighttime wakefulness, preventing relaxation and thereby exacerbating sleep difficulties (De France and Hollenstein 2019; Killgore et al. 2023; Zoccola et al. 2009). High levels of rumination are strongly associated with more severe insomnia symptoms, indicating a central role for rumination in the maintenance of sleep disturbances (Levenson et al. 2015). Furthermore, repetitive thought patterns, including both worry and rumination, have been linked to elevated nocturnal cortical arousal and altered REM sleep physiology, potentially contributing to increased sleep fragmentation and reduced sleep efficiency (SE) (Dressle et al. 2023; Galbiati et al. 2020; Kalmbach et al. 2020). Despite the well‐documented associations between emotional dysregulation, worry, rumination and insomnia, the precise nature of their interplay remains unclear. While some research suggests that emotional dysregulation may predispose individuals to excessive worry and rumination, which in turn contribute to insomnia, other studies propose a bidirectional relationship in which sleep disturbances exacerbate emotional and cognitive vulnerabilities (Kalmbach et al. 2021; Meneo et al. 2023; Palagini et al. 2017; Vandekerckhove and Wang 2018). Understanding how these factors interact is crucial for developing targeted interventions for insomnia, as addressing emotional dysregulation and maladaptive cognitive patterns may enhance the effectiveness of existing treatments.

Given the high prevalence and significant consequences of insomnia disorder, further investigation into these psychological mechanisms is essential. The present study aims to clarify the relationships between emotional dysregulation, repetitive negative thinking (worry and rumination) and REM sleep instability in individuals with insomnia, examining how these factors may contribute to the functional impact of sleep difficulties.

We hypothesise that rumination may be more strongly associated with the functional impact of insomnia, given its close link with depressive symptoms, which are frequently comorbid with insomnia. Particular attention will also be given to a specific component of emotion dysregulation, namely difficulties in distracting from emotions, as it may be closely related to repetitive negative thinking and impaired attentional disengagement.

This focus was determined a priori and is grounded in both theoretical and empirical evidence. In our previous study (Galbiati et al. 2020), several dimensions of emotion dysregulation were found to be associated with alterations in REM sleep microstructure, including a specific relationship between the ‘difficulties in distracting with emotions and performing alternate behaviour’ subscale and REM density. These results suggested that reduced attentional disengagement from emotional stimuli may be linked to REM sleep regulation and instability. Building upon this evidence, we selected this subscale as a key indicator of emotional rigidity and attentional inflexibility, processes conceptually related to repetitive negative thinking (Aldao et al. 2016; Joormann and Gotlib 2010). Furthermore, in line with our aim to investigate the functional consequences of these processes, we focused on the ‘impact’ factor of the Insomnia Severity Index (ISI), which reflects the daytime consequences and subjective burden of insomnia rather than its nocturnal symptoms. This dimension better captures the emotional distress and perceived daytime impairment associated with poor sleep quality (Castronovo et al. 2016), aligning with our hypothesis that emotional dysregulation and repetitive negative thinking contribute to the perceived daytime impact of insomnia.

The present work, a secondary analysis of the dataset reported in Galbiati et al. (2020), extends these findings by applying novel mediation models to examine the interplay between emotional dysregulation, repetitive thought and REM instability contributing to insomnia and its daytime consequences.

## 2 Methods

### 2.1 Participants

Twenty‐three patients with insomnia disorder, diagnosed by a neurologist specialised in sleep medicine according to standard criteria (American Academy of Sleep Medicine 2024), and 23 age‐ and sex‐matched healthy sleepers were enrolled at the Sleep Disorder Center of San Raffaele Hospital in Milan, Italy. The presence of dementia, psychiatric or other sleep disorders, and neurological comorbidities was considered exclusion criteria. Furthermore, patients had to be drug‐free for at least 2 months before their inclusion in the study. Healthy sleepers were enrolled based on an ISI score < 8 (Castronovo et al. 2016), an Epworth Sleepiness Scale (ESS) score < 10 (Johns 1991) and a Pittsburgh Sleep Quality Index (PSQI) score < 5 (Curcio et al. 2013) to exclude the presence of insomnia disorder, excessive daytime sleepiness or any other sleep disorders, respectively. All participants underwent a comprehensive assessment evaluating emotion dysregulation, sleep quality, insomnia severity, excessive daytime sleepiness, worry, rumination, depressive and anxious symptomatology. As part of the study protocol, only participants with insomnia disorder underwent overnight polysomnography (PSG), while healthy controls completed only the questionnaire‐based assessment. All participants provided written informed consent prior to their inclusion in the study, in accordance with the protocol approved by the local ethics committee.

### 2.2 Polysomnography

As mentioned above, only patients with insomnia disorder underwent an in‐laboratory PSG recording. The procedure has been described in detail in our related paper (Galbiati et al. 2020). Briefly, patients had to be drug naïve or without any medication for the 2 months preceding the evaluation to undergo the PSG study. Three electroencephalographic channels (F4, C4 and O2) referred to the contralateral mastoid, electrooculogram (EOG) and electromyogram (EMG) were considered for sleep scoring according to standard criteria on 30‐s epochs (Berry et al. 2012). Standard macrostructural indices were extracted: SL, total sleep time (TST), wake after sleep onset (WASO), SE, non‐REM (NREM) Sleep Stage 1 (N1), Stage 2 (N2), Slow Wave Sleep or Sleep Stage 3 (N3) and REM sleep. Arousal indices were computed according to AASM criteria (American Academy of Sleep Medicine 2024). Specifically, arousals were identified based on these scoring rules and were included in the REM arousal index only when they occurred within epochs classified as REM sleep. Arousals appearing in wake epochs immediately following REM were not counted, to ensure consistency with standard REM‐specific scoring conventions and to avoid double classification of arousal events. Furthermore, we computed a REM arousal index to evaluate the impact of emotional regulation and repetitive thought on REM sleep disruption. An arousal during REM sleep was defined as an abrupt shift of EEG frequency, including alpha, theta, and/or frequencies > 16 Hz (but not spindles) that lasts at least 3 s, with at least 10 s of stable sleep preceding the event accompanied by concurrent increases in submental EMG amplitude lasting at least 1 s (Berry et al. 2015). The arousal index was calculated as a ratio of the total arousal events over the duration of REM sleep in hours.

### 2.3 Questionnaires

#### 2.3.1 Difficulties in Emotion Regulation Scale

The Difficulties in Emotion Regulation Scale (DERS) is a self‐report measure developed to assess clinically relevant emotion dysregulation (Gratz and Roemer 2004; Sighinolfi et al. 2010). The DERS consists of 36 items, each rated on a 5‐point Likert‐type scale ranging from 1 (*almost never*) to 5 (*almost always*). Scores range from a minimum of 36 to a maximum of 180, and higher scores reflect greater difficulties in regulating emotion. Six subscales have been identified: (1) ‘Non‐acceptance of Emotional Responses’ reflects difficulties in accepting negative emotions and a tendency to experience secondary negative reactions (e.g., shame, anger or frustration) in response to one's distress; (2) ‘Difficulties in Distracting with Emotion and Performing Alternate Behaviour’ indicates problems diverting attention away from negative emotions and reduced ability to engage in goal‐directed behaviours while emotionally distressed; (3) ‘Lack of Confidence in Emotional Regulation Skills’ describes low confidence in one's ability to effectively manage, modulate or cope with negative emotional states; (4) ‘Difficulties in Behavioural Control’ reflects the difficulty in maintaining control over personal behaviours when experiencing negative emotions; (5) ‘Difficulty in Recognising Emotions’ indicates a reduced ability to identify, label or differentiate one's emotional experiences; (6) ‘Reduced Emotional Self‐Awareness’ reflects limited attention or awareness towards one's own emotions and internal affective states (Sighinolfi et al. 2010). *α* = 0.91.

#### 2.3.2 ISI

ISI is a questionnaire that evaluates the severity of insomnia symptoms on a 5‐point Likert scale of 0–4 for each of the seven items. The total score ranges from 0 to 28, with higher scores indicating greater insomnia severity, and is divided as follows: 0–7 no significant insomnia; 8–14 subthreshold insomnia; 15–21 moderate insomnia and 22–28 severe insomnia. Three different factors named ‘severity’ (ISI1a, ISI1b, ISI1c), ‘impact’ (ISI3, ISI4, ISI5) and ‘satisfaction’ (ISI5, ISI2, ISI1a) have been identified. The severity factor reflects the intensity of core insomnia symptoms such as difficulty falling or staying asleep; the impact factor assesses the perceived consequences of insomnia on daily functioning and quality of life; and the satisfaction factor captures the individual's overall subjective satisfaction or dissatisfaction with their sleep (Castronovo et al. 2016). *α* = 0.75.

#### 2.3.3 Penn State Worry Questionnaire (PSWQ)

PSWQ is a 16‐item instrument aimed at measuring the trait of worry. A total score of 50 has been identified as a reliable cut‐off to discriminate generalised anxiety disorder patients from healthy subjects (Meyer et al. 1990; Stanley et al. 2003). *α* = from 0.88 to 0.95.

#### 2.3.4 Ruminative Response Scale (RRS)

The RRS includes 22 items characterising responses to depressed moods that are divided into three types: self‐focused responses, symptoms‐focused responses and responses focused on the possible consequences and causes of the mood. Every item has to be rated on a scale from 1 (*almost never*) to 4 (*almost always*) (Nolen‐Hoeksema et al. 1999; Palmieri et al. 2007). *α* = from 0.71 to 0.80.

#### 2.3.5 Beck Depression Inventory (BDI)

The BDI is a self‐report inventory composed of 21 items aimed to assess depressive symptomatology. Each item is scored on a 0–3 value. According to the total score, it is possible to identify 0–13: minimal depression; 14–19: mild depression; 20–28: moderate depression and 29–63: severe depression (Beck et al. 1996). *α* = 0.89.

#### 2.3.6 The State–Trait Anxiety Inventory (STAI)

The STAI provides a measure of trait‐ and state‐anxiety. In this version (STAI‐Y), the inventory is composed of two scales, STAI‐Y‐1 and STAI‐Y‐2, that can be used independently. STAI‐Y‐1 consists of 20 items that assess how the subject feels ‘at this moment’. STAI‐Y‐2 is composed of 20 items evaluating how the subject feels ‘generally’. For both scales, the total score ranges from 20 to 80. Higher scores indicate higher levels of anxiety (Tenenbaum et al. 1985). *α* = from 0.86 to 0.95.

### 2.4 Statistical Analysis

Given the secondary and correlational nature of the dataset, all analyses were considered exploratory and hypothesis‐generating. Statistical analyses were conducted using JASP 0.18.3. Descriptive statistics were computed to summarise sample characteristics, and Spearman correlation coefficients were performed to examine relationships between emotional dysregulation (DERS), worry (PSWQ), rumination (RRS), insomnia severity (ISI) and depressive symptoms (BDI). In addition, we examined the association between worry (PSWQ) and rumination (RRS) to better characterise their interrelationship, given their conceptual overlap as forms of repetitive negative thinking. Mann–Whitney *U* tests were used to compare differences between insomnia patients and healthy controls on key variables.

In order to test our hypotheses, we conducted three separate mediation models using maximum likelihood estimation and bias‐corrected bootstrapping (1000 resamples) to assess the significance of indirect effects. In all models, mediation was considered significant if the 95% confidence interval (CI) for the indirect effect did not include zero.

Model 1 examined whether rumination (RRS) and worry (PSWQ) mediated the relationship between overall emotional dysregulation (total DERS score) and the impact of insomnia (ISI subscale).

Model 2 explored whether rumination (RRS) and worry (PSWQ) mediated the relationship between the DERS subscale ‘Difficulties in Distracting with Emotions’ and depressive symptoms (BDI total score). Model 3 tested a parallel mediation model in which the same DERS subscale (‘Difficulties in Distracting with Emotions’) predicted the impact of insomnia (ISI subscale), with rumination (RRS) and the REM arousal index (REM sleep instability) as parallel mediators. For each model, we estimated the direct effect (*c*'), the total effect (*c*) and the indirect effect(s) (*a* × *b*). Standardised path coefficients, standard errors and *z*‐values were reported. Visual representations of the models are provided in Figures 4, 5, 6. All statistical tests were two‐tailed with an alpha level set at *p* < 0.05. Effect sizes were interpreted following Cohen's (1988) guidelines, with *r* = 0.10 considered small, *r* = 0.30 moderate and *r* = 0.50 large. Data were inspected for normality and homoscedasticity, and no significant violations of statistical assumptions were observed.

## 3 Results

Results regarding the comparison between patients affected by insomnia disorder and healthy sleepers have been previously reported in our previous study (Galbiati et al. 2020). In brief, insomnia patients had a significantly higher ISI score (19.65 ± 4.48) compared to healthy sleepers (3.30 ± 3.42, *U* = 2.000, *p* < 0.001). Higher levels of worry (PSWQ: 49.70 ± 12.18 vs. 38.26 ± 6.93, *U* = 111.000, *p* < 0.001), rumination (RRS: 43.91 ± 11.81 vs. 31.09 ± 6.79, *U* = 84.500, *p* < 0.001), depressive symptoms (BDI: 13.17 ± 9.09 vs. 1.78 ± 3.11, *U* = 32.500, *p* < 0.001) and anxiety (STAI‐Y1 and Y2: 43.35 ± 11.83 vs. 30.43 ± 6.86, *U* = 90.500, *p* < 0.001; 45.22 ± 11.83 vs. 31.82 ± 5.45, *U* = 62.000, *p* < 0.001) than healthy controls. Moreover, insomnia patients scored significantly higher on the DERS (79.83 ± 25.81 vs. 56.22 ± 11.65, *U* = 108.500, *p* < 0.001). Regarding the DERS subscales, patients exhibited significantly higher scores on ‘Non‐Acceptance of Emotional Responses’ (*U* = 127.5000, *p* < 0.001), ‘Difficulties in Distracting with Emotion and Performing Alternate Behaviour’ (*U* = 113.500, *p* < 0.001), ‘Lack of Confidence in Emotional Regulation Skills’ (*U* = 76.000, *p* < 0.001), ‘Difficulties in Behavioural Control’ (*U* = 87.000, *p* < 0.001) and ‘Difficulties in Recognising Emotions’ (*U* = 145.000, *p* < 0.05), except for ‘Reduced Emotional Self‐Awareness’, which did not differ significantly.

**FIGURE 1:**
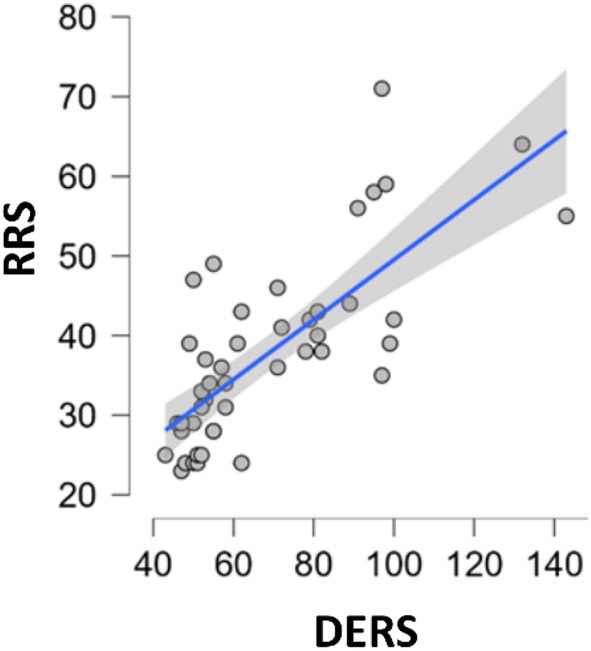
Association between emotion dysregulation and rumination. DERS, Difficulties in Emotion Dysregulation Scale; RRS, Ruminative Response Scale.

Correlational analyses showed a significant association between DERS and RRS, *r* = 0.727, *p* < 0.001 (Figure 1) and with all its subscales, ‘depression’ *r* = 0.781, *p* < 0.001, ‘reflections’ *r* = 0.605, *p* < 0.001 and ‘brooding’ *r* = 0.683, *p* < 0.001.

**FIGURE 2:**
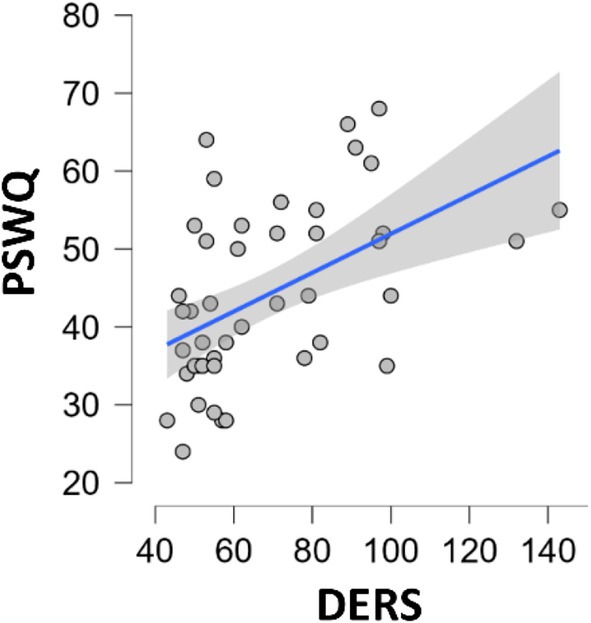
Association between emotion dysregulation and worry. DERS, Difficulties in Emotion Dysregulation Scale; PSWQ, Penn State Worry Questionnaire.

A positive association was also found between PSWQ and DERS (*r* = 0.528, *p* < 0.001) (Figure 2), but with a smaller effect in comparison to RRS and its subscale.

**FIGURE 3:**
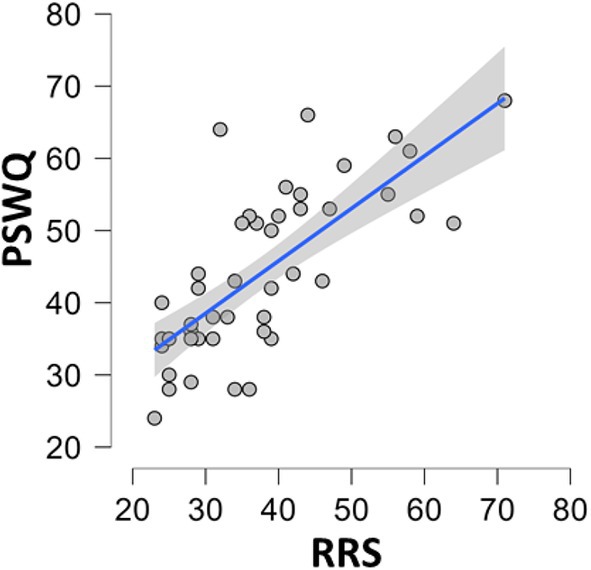
Association between worry (PSWQ) and rumination (RRS). PSWQ, Penn State Worry Questionnaire; RRS, Ruminative Response Scale.

Moreover, a significant positive correlation was observed between worry and rumination (*r* = 0.767, *p* < 0.001) (Figure 3). This association highlights their conceptual overlap as forms of repetitive negative thinking, while also indicating that they capture partially distinct dimensions of cognitive–emotional processing.

Interestingly, only the DERS subscale ‘Difficulties in distracting with emotions and performing alternative behaviour’ had a significant positive association with the REM arousal index (*r* = 0.531, *p* < 0.005) evaluated only in the sample of patients affected by insomnia (see Galbiati et al. 2020 for the original figure).

### 3.1 Mediation Analysis

We tested a mediation model examining the relationships between emotional dysregulation, worry, rumination and the impact of insomnia, a subscale of the ISI evaluating the extent to which insomnia symptoms interfere with daily functioning (Castronovo et al. 2016). The direct effect of emotional dysregulation on the impact of insomnia was not statistically significant (estimate = 0.032, SE = 0.028, *z* = 1.148, *p* = 0.251, 95% CI [−0.022, 0.086]).

For the indirect effects, emotional dysregulation affected the impact of insomnia via rumination (estimate = 0.077, SE = 0.028, *z* = 2.713, *p* = 0.007, 95% CI [0.021, 0.133]), suggesting a significant mediating role. However, the indirect effect through worry was not significant (estimate = −0.002, SE = 0.014, *z* = −0.166, *p* = 0.868, 95% CI [−0.029, 0.024]). The total indirect effect of emotional dysregulation on the impact of insomnia was statistically significant (estimate = 0.075, SE = 0.023, *z* = 3.244, *p* < 0.001, CI [0.030, 0.120]), indicating that mediation primarily occurred through rumination.

The total effect of emotional dysregulation on the impact of insomnia was also significant (estimate = 0.107, SE = 0.020, *z* = 5.214, *p* < 0.001, 95% CI [0.066, 0.147]), suggesting an overall impact despite the non‐significant direct effect. Additionally, path coefficients indicated that emotional dysregulation significantly predicted rumination (estimate = 0.376, SE = 0.048, *z* = 7.786, *p* < 0.001, 95% CI [0.281, 0.470]) and worry (estimate = 0.249, SE = 0.063, *z* = 3.972, *p* < 0.001, 95% CI [0.126, 0.371]). However, only rumination significantly predicted the impact of insomnia (estimate = 0.205, SE = 0.071, *z* = 2.894, *p* = 0.004, 95% CI [0.066, 0.344]), whereas worry did not (estimate = −0.009, SE = 0.055, *z* = −0.166, *p* = 0.868, 95% CI [−0.116, 0.098]). A significant residual covariance was found between worry and rumination (estimate = 45.534, SE = 12.647, *z* = 3.600, *p* < 0.001, 95% CI [20.745, 70.322]), indicating shared variance between these constructs.

**FIGURE 4:**
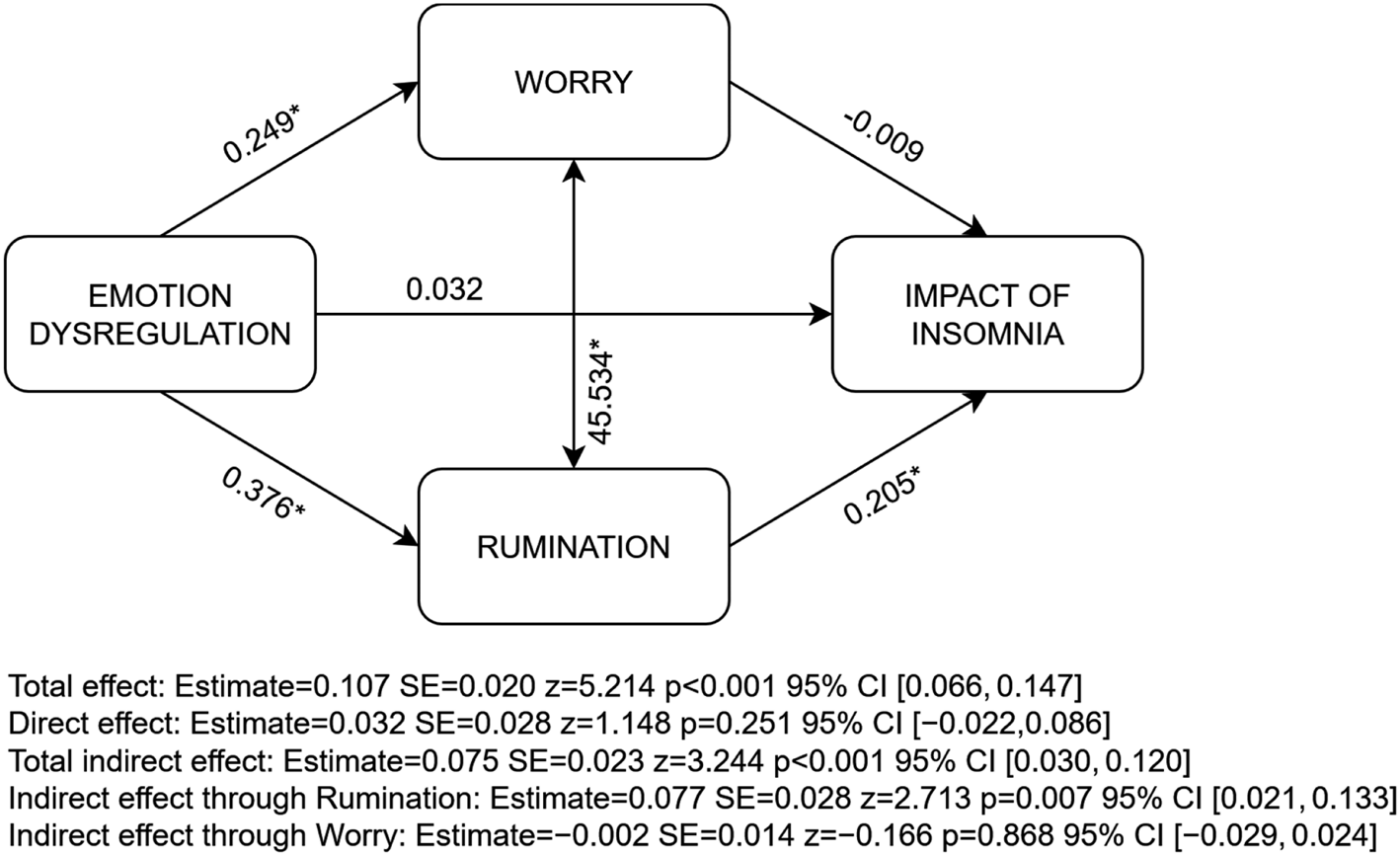
Mediation model evaluating the impact of emotion dysregulation on the impact of insomnia through worry and rumination.

These findings suggest that the relationship between emotional dysregulation and insomnia‐related impact is mediated by rumination, whereas worry does not play a significant mediating role (see Figure 4).

As a second model, we examined the relationship between the DERS subscale ‘Difficulties in Distracting with Emotions’ and depressive symptoms, considering the mediating roles of worry and rumination.

The direct effect of Difficulties in Distracting with emotions on depressive symptoms was significant (estimate = 0.837, SE = 0.194, *z* = 4.312, *p* < 0.001, 95% CI [0.456, 1.217]), indicating a strong association.

Regarding indirect effects, the mediation through rumination was significant (estimate = 0.478, SE = 0.188, *z* = 2.537, *p* = 0.011, 95% CI [0.109, 0.846]), suggesting that rumination partially explains the link between difficulties in distracting with emotions and depression. In contrast, the mediation via worry was not significant (estimate = −0.030, SE = 0.095, *z* = −0.315, *p* = 0.753, 95% CI [−0.216, 0.156]), indicating that worry does not contribute meaningfully to this relationship.

The total indirect effect was significant (estimate = 0.448, SE = 0.149, *z* = 3.006, *p* = 0.003, 95% CI [0.156, 0.740]), reinforcing the mediating role of rumination in the association between ‘Difficulties in Distracting with Emotions’ subscale and depressive symptoms. Additionally, the total effect was significant (estimate = 1.285, SE = 0.163, *z* = 7.894, *p* < 0.001, 95% CI [0.966, 1.603]), confirming a strong overall relationship between these variables.

Path coefficients showed that ‘Difficulties in Distracting with Emotions’ significantly predicted rumination (estimate = 1.459, SE = 0.244, *z* = 5.986, *p* < 0.001, 95% CI [0.982, 1.937]) and worry (estimate = 0.956, SE = 0.288, *z* = 3.313, *p* < 0.001, 95% CI [0.390, 1.521]), but only rumination significantly predicted depressive symptoms (estimate = 0.327, SE = 0.117, *z* = 2.801, *p* = 0.005, 95% CI [0.098, 0.556]), whereas worry did not (estimate = −0.031, SE = 0.099, *z* = −0.317, *p* = 0.752, 95% CI [−0.225, 0.162]).

Additionally, a significant residual covariance was observed between worry and rumination (estimate = 57.151, SE = 15.274, *z* = 3.742, *p* < 0.001, 95% CI [27.215, 87.087]), suggesting shared variance between worry and rumination.

**FIGURE 5:**
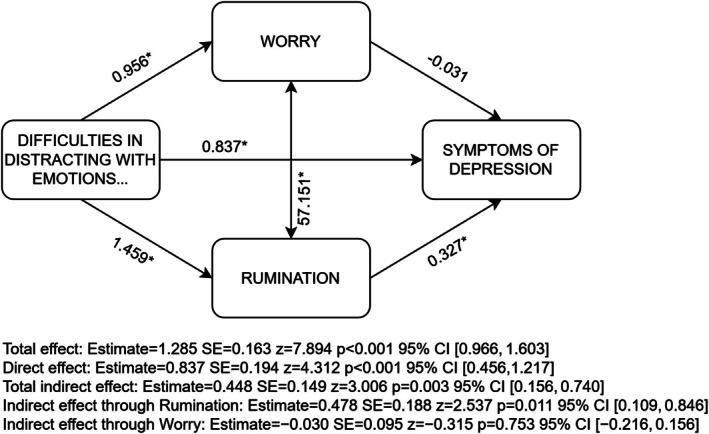
Mediation model evaluating the impact of difficulties in distracting with emotions on the impact of depressive symptoms through worry and rumination.

These findings suggest that rumination, but not worry, mediates the relationship between difficulties in distraction with emotions and depressive symptoms (see Figure 5).

A last mediation analysis was conducted only in the sample of patients affected by insomnia to examine the role of the REM Arousal Index in mediating the relationship between ‘Difficulties in Distracting with Emotion and Performing Alternate Behaviour’ and the impact of insomnia. The direct effect of ‘Difficulties in Distracting with Emotions’ on the impact of insomnia was not statistically significant (estimate = 0.036, SE = 0.116, *z* = 0.308, *p* = 0.758, 95% CI [−0.192, 0.264]). This suggests no direct prediction.

Mediation analysis revealed significant indirect effects. Specifically, Difficulties in Distracting with emotions exerted a significant indirect effect on the impact of insomnia through rumination (estimate = 0.238, SE = 0.078, *z* = 3.057, *p* = 0.002, 95% CI [0.085, 0.391]). Similarly, an indirect effect was observed through the REM arousal index (estimate = 0.204, SE = 0.099, *z* = 2.063, *p* = 0.039, 95% CI [0.010, 0.398]). These results indicate that the relationship between ‘Difficulties in Distracting with emotions’ and the impact of insomnia is mediated by rumination and REM Arousal Index.

The total effect of Difficulties in Distracting with emotion on the impact of insomnia was statistically significant (estimate = 0.478, SE = 0.090, *z* = 5.332, *p* < 0.001, 95% CI [0.302, 0.654]). This indicates that when considering both direct and indirect effects, ‘Difficulties in Distracting with Emotions’ significantly influences the impact of insomnia.

The combined indirect effects of ‘Difficulties in Distracting with Emotions’ on the impact of insomnia were also significant (estimate = 0.442, SE = 0.111, *z* = 3.979, *p* < 0.001, 95% CI [0.224, 0.660]), further supporting the role of rumination and REM arousal index as mediators.

The residual covariance between rumination and REM arousal index was not statistically significant (estimate = 15.080, SE = 21.878, *z* = 0.689, *p* = 0.491, 95% CI [−27.800, 57.960]). This indicates that rumination and REM arousal index do not share substantial unexplained variance beyond the effects already modelled.

**FIGURE 6:**
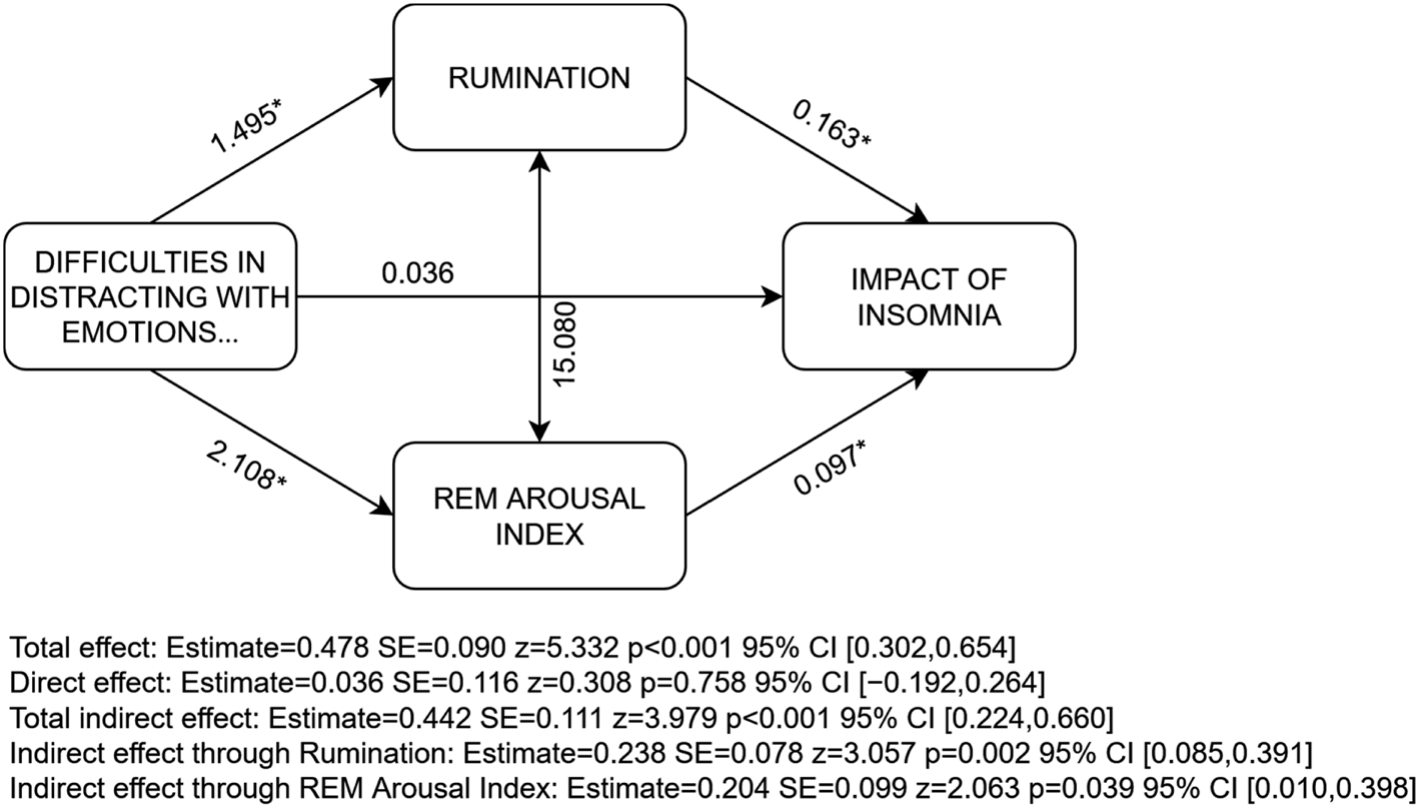
Mediation model evaluating the impact of difficulties in distracting with emotions on the impact of insomnia through rumination and REM arousal index.

Overall, the findings suggest that while ‘Difficulties in Distracting with Emotions’ does not directly predict the impact of insomnia, it exerts a significant indirect influence through rumination and REM arousal index. These mediators play a crucial role in linking cognitive distraction difficulties to insomnia consequences, emphasising the importance of emotion regulation and physiological arousal in this relationship (see Figure 6).

### 3.2 Sensitivity Analyses

To further examine the robustness and specificity of our main mediation models, additional analyses were conducted for each DERS subscale. Two parallel sets of mediation models were computed: (1) models using depressive symptoms (BDI) as the outcome and rumination (RRS) and worry (PSWQ) as mediators and (2) models using the ISI impact subscale as the outcome and rumination (RRS) and REM arousal index as mediators. All models were estimated using the same analytic procedures described above, including bootstrapped confidence intervals for indirect effects. The results are reported in Tables S1.1 and S1.2. Overall, the pattern of findings confirmed the robustness of the primary results: across both sets of models, rumination consistently mediated the link between emotional dysregulation and both depressive and insomnia‐related outcomes, while worry and REM arousal showed weaker or inconsistent mediation effects.

To address potential confounding effects due to diagnostic heterogeneity, we re‐ran the correlational and mediation analyses including only participants with insomnia disorder. Within this group, the correlation between emotional dysregulation (DERS) and rumination (RRS) remained significant (*r* = 0.698, *p* < 0.001), whereas the correlation between DERS and worry (PSWQ) was not significant. We also repeated the first two mediation models restricted to the insomnia group. The first model (outcome = ISI impact) remained significant for the indirect path through rumination (*p* = 0.011), while the indirect effect through worry was not significant, replicating the main finding observed in the full sample. In contrast, the second model (outcome = BDI) did not reach statistical significance when limited to the insomnia group. Taken together, these results partially replicate the original pattern, indicating that the mediation effect through rumination remains significant and specific within the insomnia group (see Tables S2.1 and S2.2).

Finally, as an additional exploratory analysis, we tested an alternative mediation model in which trait anxiety (STAI‐Y2) was included as the outcome variable instead of depressive symptoms (BDI). This analysis aimed to examine whether the observed relationships between the DERS subscale ‘Difficulties in Distracting with Emotions’, rumination and worry extended to anxiety‐related outcomes. In this additional model, trait anxiety (STAI‐Y2) was chosen as the outcome variable because ‘Difficulties in Distracting with Emotions’, rumination and worry represent relatively stable, trait‐like tendencies rather than transient states, making this measure conceptually more consistent with the other constructs examined. The model yielded conceptually consistent results, with rumination emerging as the main mediator linking Difficulties in Distracting with Emotions to trait anxiety. This analysis is reported in Figure S3.

## 4 Discussion

The present study provides novel insights into the interplay between emotional dysregulation, worry, rumination and their impact on insomnia disorder and depressive symptoms. Our findings highlight the role of rumination in mediating the effects of emotional dysregulation on sleep disturbances and depression, whereas worry appears to have a less significant impact. Moreover, we identified an association between ‘Difficulties in distracting with emotions’ component of emotional dysregulation and REM sleep instability, further emphasising the importance of cognitive and emotional processes in insomnia disorder.

### 4.1 The Mediating Role of Rumination in Insomnia

Our mediation analysis confirmed that emotional dysregulation significantly influences the functional impact of insomnia through rumination. This finding aligns with previous research indicating that rumination is associated with prolonged SL and increases nocturnal awakenings by sustaining cognitive arousal before sleep (Kalmbach, Anderson, et al. 2018; Kalmbach, Cuamatzi‐Castelan, et al. 2018; Levenson et al. 2015). Individuals with insomnia often engage in repetitive negative thoughts about their sleep difficulties, further exacerbating hyperarousal and sleep fragmentation (Carney et al. 2013; Palagini et al. 2017). The significant indirect effect of emotional dysregulation on the daytime consequences of insomnia via rumination suggests that targeting rumination through cognitive‐behavioural interventions may be a promising approach for improving sleep quality in individuals with insomnia.

Conversely, the indirect effect of emotional dysregulation on insomnia through worry was not significant. While worry is known to contribute to sleep disturbances (Gerlach et al. 2020; Nota and Coles 2018), our findings suggest that its impact may be less pronounced when compared to rumination. This aligns with evidence indicating that rumination is associated with reduced SE, increased WASO, and overall poorer sleep quality, whereas worry does not show the same effects on sleep parameters (Carney et al. 2010). These findings, together with the correlational results, support the notion that both rumination and worry are significantly associated with emotional dysregulation and can be conceptualised as forms of repetitive dysfunctional thinking. Both are characterised by abstract, verbal and intrusive thought patterns that are difficult to control and contribute to the persistence of emotional distress (Borkovec et al. 1983; Wahl et al. 2019). Despite these shared features, rumination and worry are conceptually distinct and exert differential effects on sleep (Carney et al. 2010). This distinction may arise from the differing temporal orientations of these cognitive processes: worry is predominantly future‐focused and centres on potential threats, whereas rumination involves a repetitive focus on past experiences and internal states, which may render it particularly detrimental to sleep by amplifying negative effects (Nolen‐Hoeksema 2000; Zhou et al. 2020). Moreover, whereas worry is also viewed as a naive goal‐directed cognitive strategy aimed at anticipating and managing potential problems, rumination reflects a maladaptive attempt to process or resolve emotional distress, often leading to a worsening of emotional dysregulation (Mansueto et al. 2024; Nolen‐Hoeksema et al. 2008). This distinction is particularly relevant in the context of insomnia, as rumination appears to contribute more directly to the cognitive and emotional hyperarousal underlying the disorder (Baglioni et al. 2010; Harvey 2002). Unlike worry, which may initially serve a problem‐solving function, rumination tends to perpetuate negative mood and self‐focused attention, both of which interfere with the ability to mentally disengage at bedtime (Espie et al. 2006). This sustained pre‐sleep arousal has been identified as a core mechanism in the development and maintenance of difficulties initiating and maintaining sleep (Espie et al. 2006; Pillai and Drake 2015). Overall, these findings underscore the importance of distinguishing between forms of repetitive negative thinking in the context of insomnia. Investigating the specific pathways through which rumination and worry influence sleep may help refine intervention strategies and improve outcomes for individuals with chronic insomnia.

### 4.2 Difficulties in Distracting With Emotions and Depressive Symptoms

The ‘Difficulties in Distracting with Emotions’ subscale of DERS measures an individual's struggle to shift attention away from distressing emotions. It assesses how effectively a person can use external activities or cognitive strategies to regulate emotions. Higher scores indicate greater difficulty in engaging in distraction‐based emotion regulation techniques (Sighinolfi et al. 2010).

The second mediation model examined the link between ‘Difficulties in Distracting with Emotions’ and depressive symptoms, revealing a significant indirect effect through rumination. This aligns with prior evidence suggesting that impairments in attentional disengagement from emotionally salient material may foster persistent, self‐focused negative thinking, thereby maintaining and exacerbating depressive affect (Joormann and Gotlib 2010; Nolen‐Hoeksema et al. 2008; Slavish and Graham‐Engeland 2015). The significant indirect effect observed through rumination is consistent with the ‘Response Styles Theory’ (Nolen‐Hoeksema 1991), which posits that individuals who ruminate in response to negative mood are more likely to experience sustained and intensified depressive states.

Although difficulties in emotional distraction were also associated with worry, worry did not significantly account for depressive symptoms in the model. This result reinforces the notion that rumination plays a more prominent role than worry in the maintenance of depression. While worry is typically linked to anxiety‐related processes, rumination, due to its passive, repetitive and past‐oriented nature, appears more strongly implicated in depressive symptomatology (Watkins and Roberts 2020). Considering the role of rumination in insomnia, as evidenced in our first mediation model, the observed association between difficulties in emotional distraction and rumination may help explain the frequent co‐occurrence of depression and insomnia. Both conditions may share a common cognitive pathway characterised by repetitive negative thinking. Notably, a growing body of literature supports a bidirectional relationship between insomnia and depression, with each condition serving as both a risk factor and a consequence of the other (Baglioni et al. 2011; Hertenstein et al. 2019). Neurobiological studies further suggest that these disorders share underlying mechanisms such as hypothalamic–pituitary–adrenal axis dysregulation, increased cortical arousal and disturbances in REM sleep architecture (Barden 2004; Buckley and Schatzberg 2005; Riemann et al. 2025). Clinically, the reciprocal link between insomnia and depression suggests that treating sleep disturbances may also alleviate mood symptoms. Targeting rumination in individuals with comorbid insomnia and depression could enhance emotional disengagement and reduce cognitive arousal, improving both sleep and mood outcomes.

### 4.3 The Role of REM Sleep Instability

An interesting finding of this study is the association between the DERS subscale ‘Difficulties in distracting with emotions’ and REM sleep instability, as indicated by the REM arousal index. This result extends previous work demonstrating that emotional dysregulation is linked to disruptions in REM sleep (Galbiati et al. 2020; Riemann et al. 2025). REM sleep is critical for emotional processing and memory consolidation, and increased REM arousal may reflect an inability to downregulate emotional responses during sleep (Palagini et al. 2018). Our findings suggest that individuals with greater difficulties in distracting from emotions may experience heightened nocturnal cortical arousal, leading to fragmented REM sleep and worsening daytime consequences of insomnia.

Interestingly, our mediation analysis indicated that the relationship between ‘Difficulties in Distracting with Emotions’ and the functional impact of insomnia was significantly mediated by both rumination and REM arousal, highlighting a dual pathway through which cognitive and physiological mechanisms interact to exacerbate sleep disturbances. REM sleep instability and rumination are closely linked in insomnia, creating a cycle that disrupts restorative sleep. Rumination, characterised by repetitive negative thoughts, heightens emotional and physiological arousal, making it harder to transition into stable REM sleep. In turn, fragmented or unstable REM sleep may impair emotional regulation, reinforcing ruminative thought patterns. This bidirectional relationship perpetuates sleep disturbances, contributing to the persistence of insomnia and its associated cognitive and emotional difficulties. These findings underscore the importance of addressing both repetitive negative thought patterns and physiological arousal in the treatment of insomnia.

### 4.4 Clinical Implications

From a clinical perspective, our results suggest that interventions targeting rumination may be particularly beneficial for individuals with insomnia and depression. Cognitive‐behavioural therapy for insomnia (CBT‐I) and mindfulness‐based interventions have shown efficacy in reducing rumination and improving sleep quality (Mao et al. 2023; Perestelo‐Perez et al. 2017). Moreover, strategies aimed at enhancing emotional regulation skills, such as acceptance‐based therapies and cognitive reappraisal, may help mitigate the impact of emotional dysregulation on sleep and mood disorders (Calkins et al. 2015; Hofmann et al. 2009).

Our finding that rumination, but not worry, mediates the relationship between emotional regulation and insomnia has important implications for CBT‐I. Given that rumination is a key cognitive process maintaining insomnia, targeting it explicitly within CBT‐I protocols could enhance treatment efficacy. A study by Ballesio et al. (2018) demonstrates that CBT‐I reduces ruminative thinking, reinforcing the idea that addressing maladaptive thought patterns beyond sleep‐specific concerns could be beneficial. Similarly, Cheng et al. (2020) highlight rumination as a mediator in the impact of digital CBT‐I on both insomnia and depression, suggesting that reducing rumination may have broader benefits beyond sleep improvement alone. Integrating specific cognitive techniques aimed at interrupting repetitive negative thinking, such as mindfulness strategies or rumination‐focused cognitive restructuring, could strengthen the long‐term effectiveness of CBT‐I and prevent relapse. Importantly, Mindfulness‐Based Therapy for Insomnia (MBT‐I) is an emerging approach that integrates mindfulness practices with cognitive and behavioral strategies to improve sleep (Ong and Sholtes 2010). Unlike traditional CBT‐I, which focuses on restructuring maladaptive thoughts and behaviours, MBT‐I emphasises nonjudgmental awareness of thoughts, emotions and bodily sensations. By fostering acceptance rather than resistance to sleep‐related difficulties, MBT‐I helps reduce arousal and rumination. Recent research reported how MBT‐I effectively reduced cognitive arousal, including rumination, which is linked to poor prognosis in insomnia treatment. The study highlights that mindfulness practices targeting metacognitive processes and decentering can alleviate these forms of cognitive arousal, thereby improving sleep outcomes (Kalmbach et al. 2023).

Additionally, given the observed link between emotional dysregulation and REM sleep instability, incorporating sleep‐focused interventions that address nocturnal hyperarousal, such as relaxation training, biofeedback and sleep restriction therapy, may further enhance treatment outcomes. Future research should explore the efficacy of integrated approaches that target both cognitive and physiological components of insomnia.

### 4.5 Limitations and Future Directions

Despite its strengths, this study has several limitations. The modest sample size limits both statistical power and generalisability, and polysomnographic data were unavailable for healthy controls. Future research should replicate these findings in larger and more representative cohorts. Moreover, the cross‐sectional design precludes causal interpretation; longitudinal approaches are required to clarify the temporal interplay between emotion dysregulation, repetitive thinking and sleep alterations. The lack of a measure of pre‐sleep arousal also prevented us from examining its potential role in these associations. Finally, given its exploratory nature, the present work should be viewed as a preliminary step. While the mediation models suggest possible psychological and neurophysiological pathways linking emotional dysregulation, repetitive negative thinking and REM sleep instability, confirmation through larger, prospective studies is warranted.

## 5 Conclusion

In conclusion, our findings highlight the pivotal role of rumination in mediating the relationship between emotional dysregulation and both insomnia and depressive symptoms. Difficulties in distracting from emotions were linked to REM sleep instability, further reinforcing the connection between emotional and physiological dysregulation in sleep disturbances. These results emphasise the need for interventions that target repetitive negative thinking and emotional regulation strategies to improve sleep and mental health outcomes. Future research should explore integrated therapeutic approaches that address both cognitive and neurophysiological mechanisms underlying insomnia and depression.

## Author Contributions

**Samantha Mombelli:** conceptualization, formal analysis, writing – original draft, writing – review and editing, data curation. **Elisabetta Fasiello:** conceptualization, writing – review and editing, investigation, data curation. **Maria Caterina Di Perri:** conceptualization, writing – review and editing. **Francesca Casoni:** writing – review and editing, investigation. **Marco Zucconi:** supervision, writing – review and editing. **Luigi Ferini‐Strambi:** supervision, writing – review and editing. **Andrea Galbiati:** conceptualization, writing – review and editing, formal analysis, data curation, supervision.

## Funding

The authors have nothing to report.

## Disclosure

The authors have nothing to report.

## Conflicts of Interest

The authors declare no conflicts of interest.

## Supporting information


**Data S1:** jsr70267‐sup‐0001‐supinfo.docx.

## Data Availability

The data that support the findings of this study are available from the corresponding author upon reasonable request.
