# Supplementary material for: The Interplay Between Emotion Dysregulation and Repetitive Thoughts in Insomnia Disorder: The Impact of Worry, Rumination and REM Sleep Instability
Source: J Sleep Res. 2025 Dec 14;35(4):e70267. doi: 10.1111/jsr.70267 (PMC13357897; doi:10.1111/jsr.70267)

SUPPLEMENTARY MATERIAL S1: DERS Subscale

Table S1.1: Mediation models for the DERS subscales predicting **depressive symptoms (BDI)** through Worry (PSWQ) and Rumination (RRS).

| DERS Subscale | Mediator | Direct Effect β (p) | Indirect Effect β (p) | 95% CI | Total Effect β (p) | Sig. Mediation |
| --- | --- | --- | --- | --- | --- | --- |
| Lack of Confidence in Emotion Regulation Skills | PSWQ | 0.999 (p < .001) | –0.115 (p = 0.216) | [–0.298, 0.067] | 1.241 (p < .001) | No |
|  | RRS | 0.999 (p < .001) | 0.357 (p = 0.014) | [0.072, 0.643] | 1.241 (p < .001) | Yes |
| Difficulties in Behavioral Control | PSWQ | 1.132 (p < .001) | –0.001 (p = 0.989) | [–0.169, 0.167] | 1.557 (p < .001) | No |
|  | RRS | 1.132 (p < .001) | 0.427 (p = 0.018) | [0.073, 0.780] | 1.557 (p < .001) | Yes |
| Difficulty Recognizing Emotions | PSWQ | 1.222 (p < .001) | 0.004 (p = 0.971) | [–0.189, 0.196] | 1.829 (p < .001) | No |
|  | RRS | 1.222 (p < .001) | 0.603 (p = 0.010) | [0.146, 1.060] | 1.829 (p < .001) | Yes |
| Reduced Emotional Self-Awareness | PSWQ | 0.898 (p = 0.004) | –0.019 (p = 0.706) | [–0.118, 0.080] | 1.224 (p = 0.007) | No |
|  | RRS | 0.898 (p = 0.004) | 0.344 (p = 0.342) | [–0.365, 1.054] | 1.224 (p = 0.007) | No |
| Non-Acceptance of Emotional Responses | PSWQ | 0.378 (p = 0.147) | –0.066 (p = 0.617) | [–0.324, 0.192] | 1.098 (p < .001) | No |
|  | RRS | 0.378 (p = 0.147) | 0.785 (p = 0.004) | [0.258, 1.312] | 1.098 (p < .001) | Yes |

Table S1.2: Mediation models for the DERS subscale predicting **insomnia impact (ISI)** through Rumination (RRS) and REM arousal index (REM AI).

|  | Mediator | Direct Effect β (p) | Indirect Effect β (p) | 95% CI | Total Effect β (p) | Sig. Mediation |
| --- | --- | --- | --- | --- | --- | --- |
| Lack of Confidence in Emotion Regulation Skills | RRS | 0.053 (p = 0.605) | 0.208 (p = 0.006) | [0.059, 0.356] | 0.410 (p = <.001) | Yes |
|  | REM AI | 0.053 (p = 0.605) | 0.149 (p = 0.065) | [-0.010, 0.307] | 0.410 (p = <.001) | No |
| Difficulties in Behavioral Control | RRS | -0.011 (p = 0.921) | 0.272 (p = 0.003) | [0.092, 0.453] | 0.430 (p = <.001) | Yes |
|  | REM AI | -0.011 (p = 0.921) | 0.169 (p = 0.065) | [-0.011, 0.348] | 0.430 (p = <.001) | No |
| Difficulty Recognizing Emotions | RRS | 0.018 (p = 0.899) | 0.311 (p = 0.005) | [0.096, 0.527] | 0.549 (p = <.001) | Yes |
|  | REM AI | 0.018 (p = 0.899) | 0.220 (p = 0.064) | [-0.013, 0.453] | 0.549 (p = <.001) | No |
| Reduced Emotional Self-Awareness | RRS | -0.108 (p = 0.445) | 0.107 (p = 0.350) | [-0.117, 0.330] | 0.036 (p = 0.877) | No |
|  | REM AI | -0.108 (p = 0.445) | 0.037 (p = 0.726) | [-0.169, 0.243] | 0.036 (p = 0.877) | No |
| Non-Acceptance of Emotional Responses | RRS | 0.015 (p = 0.897) | 0.286 (p = 0.009) | [0.072, 0.500] | 0.432 (p =<.001) | Yes |
|  | REM AI | 0.015 (p = 0.897) | 0.131 (p = 0.087) | [-0.019, 0.282] | 0.432 (p =<.001) | No |

SUPPLEMENTARY MATERIAL S2: Mediation analyses restricted to the insomnia group.

Table S2.1: Mediation models for the DERS subscales predicting **Insomnia impact** through Worry (PSWQ) and Rumination (RRS).

| Predictor | Mediator | Direct Effect β (p) | Indirect Effect β (p) | 95% CI | Total Effect β (p) | Sig. Mediation |
| --- | --- | --- | --- | --- | --- | --- |
| DERS | PSWQ | -0.011 (p = 0.701) | –0.019 (p = 0.174) | [–0.047, 0.008] | 0.044 (p = 0.053) | No |
|  | RRS | -0.011 (p = 0.701) | 0.074 (p = 0.011) | [0.017, 0.131] | 0.044 (p = 0.053) | Yes |

Table S2.2: Mediation models for the DERS subscale “Difficulties in Distracting with emotions” predicting **depressive symptoms (BDI)** through Worry (PSWQ) and Rumination (RRS).

| Predictor, DERS subscale | Mediator | Direct Effect β (p) | Indirect Effect β (p) | 95% CI | Total Effect β (p) | Sig. Mediation |
| --- | --- | --- | --- | --- | --- | --- |
| Difficulties in Distracting with emotions | PSWQ | 0.777 (p = 0.005) | –0.054 (p = 0.517) | [–0.216, 0.109] | 1.013 (p = <.001) | No |
|  | RRS | 0.777 (p = 0.005) | 0.290 (p = 0.182) | [-0.135, 0.715] | 1.013 (p = <.001) | No |

SUPPLEMENTARY MATERIAL S3: Mediation analyses with anxiety as an outcome.

Figure S3.1. Mediation model for the DERS subscale “Difficulties in Distracting with Emotions” predicting trait anxiety symptoms (STAI-Y2) through worry (PSWQ) and rumination (RRS).


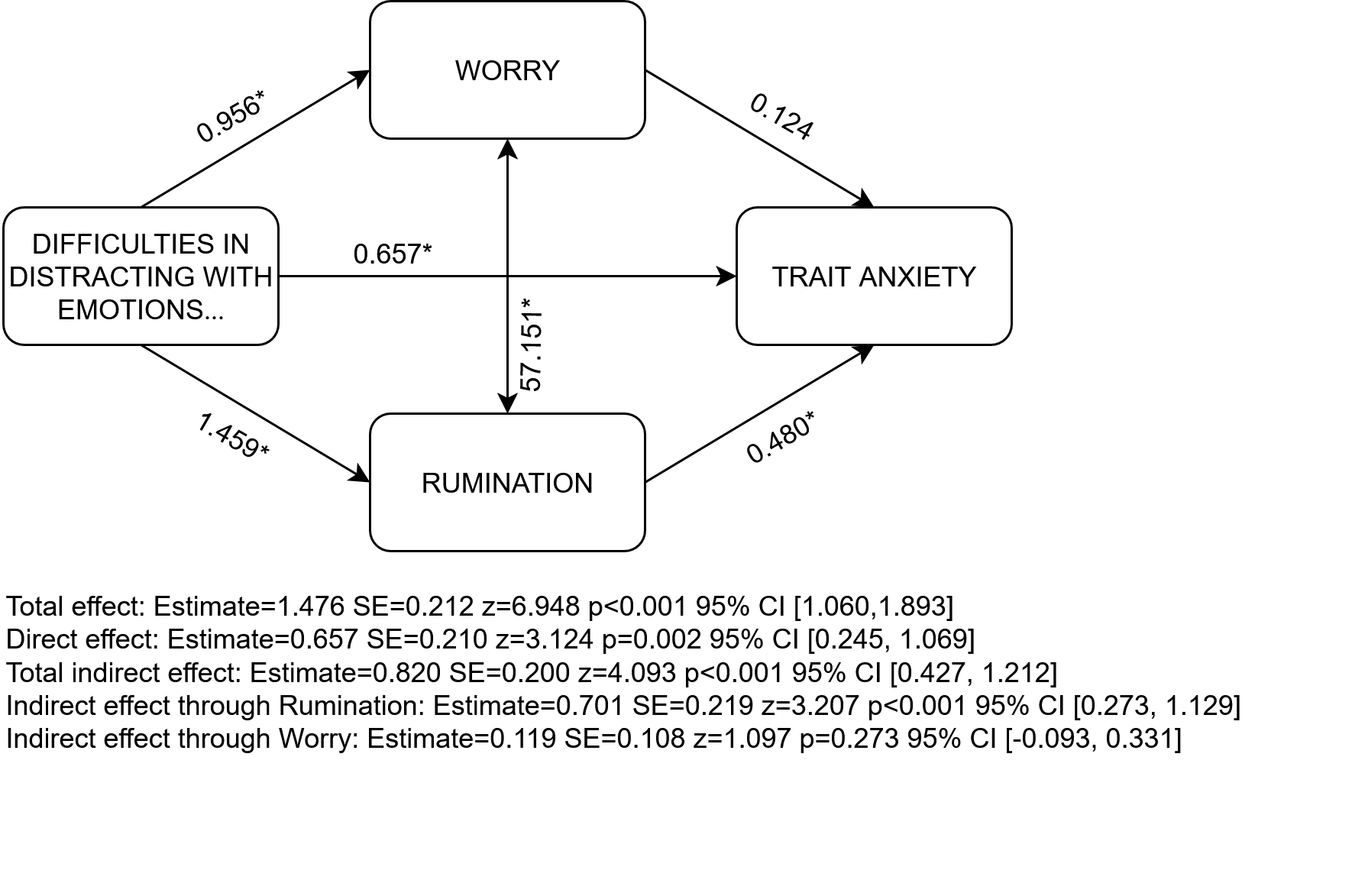

Supplement: Supplementary file 1 — Data S1: jsr70267‐sup‐0001‐supinfo.docx. [file JSR-35-e70267-s001.docx]
